# Effect of Thinning on Tree Differentiation, Productivity and Carbon Stocks of *Cryptomeria japonica* Plantations

**DOI:** 10.1002/ece3.71418

**Published:** 2025-05-07

**Authors:** Kaili Liu, Boyao Chen, Pu Zhou, Bin Zhang, Ruihui Wang, Chunsheng Wang

**Affiliations:** ^1^ Central South University of Forestry & Technology Changsha China; ^2^ Hunan Academy of Forestry Changsha China; ^3^ Yongzhou Normal College Yongzhou China; ^4^ Research Institute of Tropical Forestry Chinese Academy of Forestry Guangzhou China

**Keywords:** carbon stock, diameter class distribution, Gini coefficient, productivity, thinning treatment

## Abstract

Stand structure affects tree efficiency for a competitive use of resources and largely determines stand productivity and carbon stocks. Consequently, research on individual size and differentiation of stand structures is critical for improving monoculture‐stand productivity and carbon stock. Here, we studied the effects of four thinning intensities (CK: 0%, T1: 20%, T2: 30%, and T3: 40%) in an experimental plantation of *Cryptomeria japonica* var. *sinensis*, and assessed the individual differentiation characteristics, diameter class‐frequency distribution, stand productivity, and carbon stocks over 6 years. The results showed that the Gini coefficient decreased with increasing thinning intensity and stand age. Self‐thinning of the *C. japonica* stands occurred even after thinning, and the self‐thinning rate was relatively high at the age of 10–13 years. For T2 and T3 treatments, the self‐thinning did not occur in the 6th year after thinning. The mean diameter of each treatment increased with increasing stand age, and the normal distribution curve of diameter class frequency gradually shifted to the right, with small changes in the CK treatment and the larger one in treatment T3. Thinning increased the large‐diameter (DBH ≥ 26 cm) timber, especially in T2 and T3 treatments. Stand volume and productivity varied with stand age, with the greatest change in stand volume observed in T3, followed by that in the CK treatment. Stand productivity at different thinning intensities generally decreased and then increased with increasing stand age. Although the carbon stock of individual trees and stand increased with time, the individual trees appeared to have an obviously increasing trend with increasing thinning intensity. The results provided important insights into the implications of designing thinning intensity and timing, and determining the tree‐size class removal to meet specific management objectives.

## Graphical abstract

Tree size differentiation, stand productivity and carbon stock of thinning were explored: The Gini coefficient decreased with increasing thinning intensity and stand age. Self‐thinning was relatively high when stands were 10–13 years old but did not occur in the 6th year after thinning in treatments T2 and T3. Higher thinning intensities had the higher frequency of large‐diameter (DBH ≥ 26 cm) timber. Stand productivity at different thinning intensities generally decreased and then increased with increasing stand age. The carbon stock of individual trees increases with time, with a clear increase occurring with increasing thinning intensity.

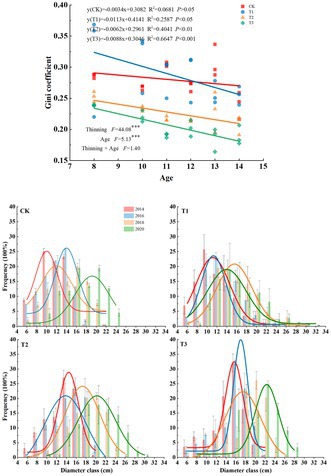

## 1 Introduction

The world's natural forest area is currently undergoing a rapid decline, with planted forests playing an important role in global forest resources. Despite China's planted forest area consistently ranking first in the world, there are still challenges, including a shortage of high‐quality large‐diameter timber and low economic and ecological benefits. The proposal of Carbon peaking and carbon neutrality goals in 2020 has led to a high demand for forest management, with monocultures being required to transition from a single pursuit of timber production to a new model of improving the quality and efficiency of ecosystem services. This change in forest management not only affects the growth of trees but also directly impacts the carbon storage and carbon sink of forests, representing a crucial means of enhancing the functionality of forest carbon sinks.

As one of the forest attributes, stand structure can be described by the degree of tree size differentiation, which determines the competitive advantage and spatial ecological niche among trees, and also reflects the stability, growth potential, and management space of stands. The influence of the stand structure on individual tree growth and development is mainly determined by the resources within the stand, their availability to the stand, and the efficiency with which the stand uses them (Wang et al. 2019; Gonçalves 2022). Among resources, those acquired and used by trees determine the individual growth rate (Binkley 2004; Bradford et al. 2010). Furthermore, resource acquisition depends on the social position of individual trees within the stand (Forrester 2019; West 2023). For example, dominant trees can exert asymmetric competition pressure on intermediate‐ and small‐path individuals through higher and larger canopies, thereby occupying a larger proportion of resources (Zambrano et al. 2019; Carvalho et al. 2022). This resource partitioning results in significantly higher resource‐use efficiency among dominant trees compared to subdominant counterparts within the stand (Binkley et al. 2010). Furthermore, resource acquisition and use efficiency are related to tree age (Binkley and Kashian 2015; Zhang et al. 2021).

Inter‐ and intraspecific competition among young trees is weak (Oheimb et al. 2011). However, as they continue to grow, canopy cover increases, canopies begin to contact and overshadow each other (Pretzsch 2021; Liu et al. 2023), whereby the intensity of the competition among trees gradually increases owing to the interlacing extension of both the aboveground crown and underground root systems (Hodge 2010; Cai et al. 2019). The differentiation among individual trees within a stand rapidly increases owing to the increasing degree of asymmetric competition with tree age, further accelerating the differentiation of individuals (Caplat et al. 2008; Lin et al. 2016), which has a significant effect on stand structure, community succession, interspecific (intraspecific) relationships, and productivity of forest ecosystems (Ratcliffe et al. 2015; Ali 2019; Ren et al. 2021). Thus, in plantation ecosystems, reducing individual differentiation within the stands is critical for cultivating large‐ and intermediate‐diameter timber stands and improving stand productivity as well as increasing stand carbon stock (Fernández Tschieder et al. 2012).

Stand density is an important factor influencing canopy structure and biomass distribution, as it can significantly alter the growth space and tree access to stand resources (Li, Liu, and Jin 2022). Usually, once individual competition within the stand reaches a certain level, the stand will naturally and gradually become increasingly sparse (Duan et al. 2019) as inferior trees die, allowing growth space and nutrients for the dominant and co‐dominant trees. Moreover, asymmetric competition for access to resources occurs much earlier in high‐density plantations (Stankova and Diéguez‐Aranda 2017). Furthermore, stand density and structure can be artificially regulated by stand management practices aimed at alleviating an extremely competitive environment encountered by stands (Sohn et al. 2016) and even reverse their effects on individual tree growth (Forrester 2014). In particular, thinning is an effective forest management measure that can reduce the degree of competition in a stand and regulate resource allocation among trees by removing some of them (Li, Barclay, et al. 2022). Thus, the remaining trees of different diameter classes will respond differently to improvements in resource availability in an environment with finite resources (Forrester 2019; Hou et al. 2019). For example, understory thinning can improve resource‐use efficiency and increase the leaf area of retained trees to increase stand yield (Moreau et al. 2020; Güney et al. 2022). To date, studies have mostly focused on the effects of tree population‐density adjustments on tree growth at the stand level (Allen et al. 2021; Kholdaenko et al. 2022), whereas less attention has been paid to the dynamics of individual growth and differentiation within stands (Pretzsch and Dieler 2011; Li et al. 2024) and the dynamic change of stand carbon stocks after thinning (Ganatsas et al. 2024).

*Cryptomeria japonica* var. *sinensis* is one of the main afforestation species in subtropical areas of China and is a fast‐growing pioneer tree in intermediate‐ and high‐altitude areas. A case in point is the western Sichuan region located in the upper reaches of the Yangtze River, where the flora is an important ecological barrier of the Yangtze River with high ecological value. The area planted with *C. japonica* reached 200,000 ha in this region. However, due to the high initial planting density and lack of effective management, the growth performance of *C. japonica* stands in this area is poor, which is not conducive to the cultivation of large‐diameter wood. Therefore, in this study, we aimed to analyze the dynamics of individual size differentiation, diameter class‐frequency distribution, and self‐thinning as well as their effects on stand productivity and stand carbon stocks, based on the long‐term monitoring experiment with different thinning treatments in an 8‐year‐old *C. japonica* plantation in western Sichuan. This study provides a theoretical basis for rational stand‐thinning to the appropriate tree density for cultivating large‐diameter wood in *C. japonica* plantations and improving stand productivity, and helps to achieve the Carbon peaking and carbon neutrality goals.

## 2 Materials and Methods

### 2.1 Experimental Site

A thinning trial in a *Cryptomeria japonica* var. *sinensis* plantation located at Yangziling Forest Farm, Ya'an City, Sichuan Province, China (29°47′37″ N, 102°56′18″ E) was established. The area has a subtropical monsoon mountain climate with abundant rainfall. The annual mean air temperature, precipitation and relatively humidity are 13.1°C, 1800 mm and 79%, respectively. The plantation was established at a spacing of 2.0 m × 1.5 m in April 2006 with an area of approximately 7 ha. *C. japonica* seedlings were raised by the Ya'an Forestry Bureau. The soil is a yellow loam with a pH value of 4.3–4.7. The contents of total nitrogen, total phosphorus, and total potassium were 2.64, 10.81, and 0.55 g·kg,^−1^ respectively. The mean elevation at the site is 1539 m above sea level, and the slope is approximately 15°.

### 2.2 Experimental Design

The thinning trial was carried out in October 2014 (the *Cryptomeria japonica* plantations were 8 years old) and was arranged in a randomized complete block design with three replications and four thinning intensity treatments, including no thinning (CK), light‐intensity thinning (T1: 20% of the trees removed), intermediate‐intensity thinning (T2: 30% of the trees removed) and strong‐intensity thinning (T3: 40% of the trees removed). Preferential felling of the diseased or crooked trees, and the smaller with poor growth trees (Table 1). Each experimental plot was 600 m^2^ and surrounded by 5 m buffer areas to reduce potential edge effects. Tree height (m) and diameter at breast height (DBH) (cm) of each tree were measured for each plot immediately after thinning (October 2014) and measured annually until October 2020. The stand density after thinning and tree growth performance of each treatment in the year 2014 and 2020 after thinning are presented in Table 2.

**TABLE 1:** Information about the removed tree among four thinning trial plantations.

Thinning intensity	Number of removed tree (trees·ha^−1^)	Mean DBH (cm)	Mean height (m)
T1	672 ± 19	8.62 ± 0.35	6.14 ± 0.18
T2	1122 ± 123	8.58 ± 0.5	6.18 ± 0.24
T3	1328 ± 63	8.81 ± 0.09	6.29 ± 0.03

*Note:* The values are the mean ± standard deviation (*n* = 3).

**TABLE 2:** Information on the thinning trial plantation.

Thinning intensity	Stand density (trees·ha^−1^)	Year 2014		Year 2020		Increase of each year	
		Mean DBH (cm)	Mean height (m)	Mean DBH (cm)	Mean height (m)	Mean DBH (cm)	Mean height (m)
CK	2833 ± 25	11.50 ± 0.30b	6.80 ± 0.10d	16.90 ± 0.10d	11.80 ± 0.66c	0.93 ± 0.01b	0.75 ± 0.11b
T1	2266 ± 44	12.37 ± 1.75b	7.17 ± 0.15c	18.10 ± 0.10c	12.30 ± 0.10c	1.05 ± 0.13ab	0.83 ± 0.03b
T2	1983 ± 85	13.27 ± 0.47ab	8.90 ± 0.30a	20.10 ± 0.35b	13.23 ± 0.21b	1.14 ± 0.05a	0.77 ± 0.03b
T3	1700 ± 69	14.27 ± 0.23a	7.80 ± 0.10b	20.77 ± 0.31a	14.10 ± 0.60a	1.07 ± 0.07ab	0.99 ± 0.09a

*Note:* The values are the mean ± standard deviation (*n* = 3), followed by different letters showing significant differences in the four thinning treatments for each index at the 0.05 probability level.

### 2.3 Statistical Analysis

#### 2.3.1 Calculation of Stand Productivity

According to the results of the annual investigation of each tree of *Cryptomeria japonica* stand, the standing volume of the living trees in the experimental plantation was calculated using the binary standing volume formula (Liu 2019):

$$V=5.611664\times 10^{-4}\times {\mathrm{DBH}}^{1.802483}H^{1.082741}$$

where: *V* is the volume per tree, m^3^; DBH is the diameter at breast height, cm; H is the height of the tree, m.

The total volume of living trees per unit area was calculated as the cumulative volume of living trees per unit area. Stand productivity was expressed as the annual increment in total tree volume per unit area per year (m^3^·ha^−1^·a^−1^).

#### 2.3.2 Characterization of the Degree of Differentiation of Individual Trees

Referring to the method of (Yang et al. 2019), the Gini coefficient was used to characterize the degree of individual size differentiation in the stand. The Gini coefficient was first proposed by Lorenz in 1905 and has since been used in the social sciences to analyze the degree of differences in income. Subsequently, the Gini coefficient was introduced to characterize the degree of individual size differentiation within plant populations and has been widely used. In this study, the Gini coefficient was calculated using the following formula:

$$\text{Gini}=1-\frac{1}{n}\sum_{i=1}^{n}\left\{\frac{\sum^{i-1}Y_{i}+\sum^{i}Y_{i}}{\sum_{i=1}^{n}Y_{i}}\right\}$$

where: *n* is the sample area inside the stocking number; *Y*_*i*_ is the basal area of living trees.

#### 2.3.3 Self‐Thinning of Stands

The self‐thinning rate of the stand was expressed as the number of dead trees in the current year divided by the total number of trees and multiplied by 100%. To determine the effect of individual dead trees on the overall stand during self‐thinning, the ratio of the mean basal area of self‐thinning individuals per year to the mean basal area before stand death (BA_death_:BA_total_) was used. BA_death_:BA_total_ > 1 indicates that the mean size of dead individual trees is larger than the stand level; BA_death_:BA_total_ < 1 indicates that the mean size of dead individual trees is smaller than the stand level.

#### 2.3.4 Diameter Classification

Using 2 cm for the class interval, the composition of the stand diameter‐class structure and its frequency were calculated based on the DBH measured for each sampled tree, and the distribution of stand diameter was plotted by using Origin 20.0, fitted with normal curves. At the same time, according to the classification of the *Cryptomeria japonica* logs, the *C. japonica* was divided into large‐diameter (DBH ≥ 26 cm), middle‐diameter (26 cm > DBH ≥ 20 cm), and small‐diameter (20 cm > DBH ≥ 6 cm).

#### 2.3.5 Carbon Stocks

We calculated the carbon stock based on the ministerial standard for *Cryptomeria japonica* (Tree biomass models and related parameters to carbon accounting for *Cryptomeria japonica* [LY/T 2657‐2016]) (Zeng 2020). The carbon stock amount of an individual tree in each study plot was calculated by the biomass values of different components multiplied by the carbon concentration of each corresponding component. The stand carbon stock was also obtained by using the carbon stock of an individual tree multiplied by the stand density.

Statistical analysis of the data was performed using Excel 2016 and SPSS 20.0. Data shown are mean ± standard deviation. Repeated measures ANOVA was used to examine the effects of thinning intensity and age on individual size differentiation, diameter class‐distribution characteristics, stand productivity, and carbon stock in the *Cryptomeria japonica* plantation. Tukey's test (*n* = 3) was used for multiple comparisons of the significance of differences at *α* = 0.05 significance level.

## 3 Result

### 3.1 Individual Size Differentiation Characteristics

**FIGURE 1:**
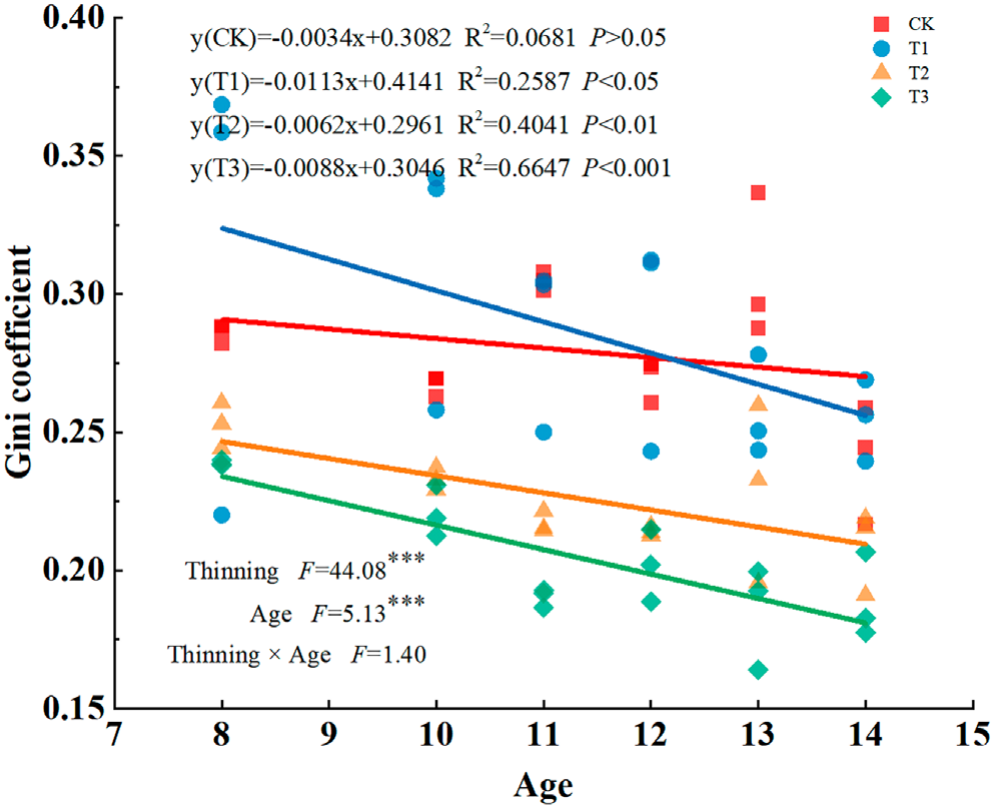
Changes in Gini coefficients of *Cryptomeria japonica* plantations with four thinning intensities. CK: No thinning, T1: Light‐intensity thinning (20% of the trees removed), T2: Intermediate‐intensity thinning (30% of the trees removed), T3: Strong‐intensity thinning (40% of the trees removed).

The Gini coefficient decreased significantly (*F* = 44.08, *p* < 0.001) with increasing thinning intensity, and the degree of individual differentiation decreased significantly (*F* = 5.13, *p* < 0.001) with increasing stand age. Meanwhile, the interaction between stand age and thinning intensity had no significant effect on the Gini coefficient (Figure 1). Furthermore, there was a significantly negative linear correlation between the Gini coefficient and stand age for all thinning intensities, except for CK treatment.

**FIGURE 2:**
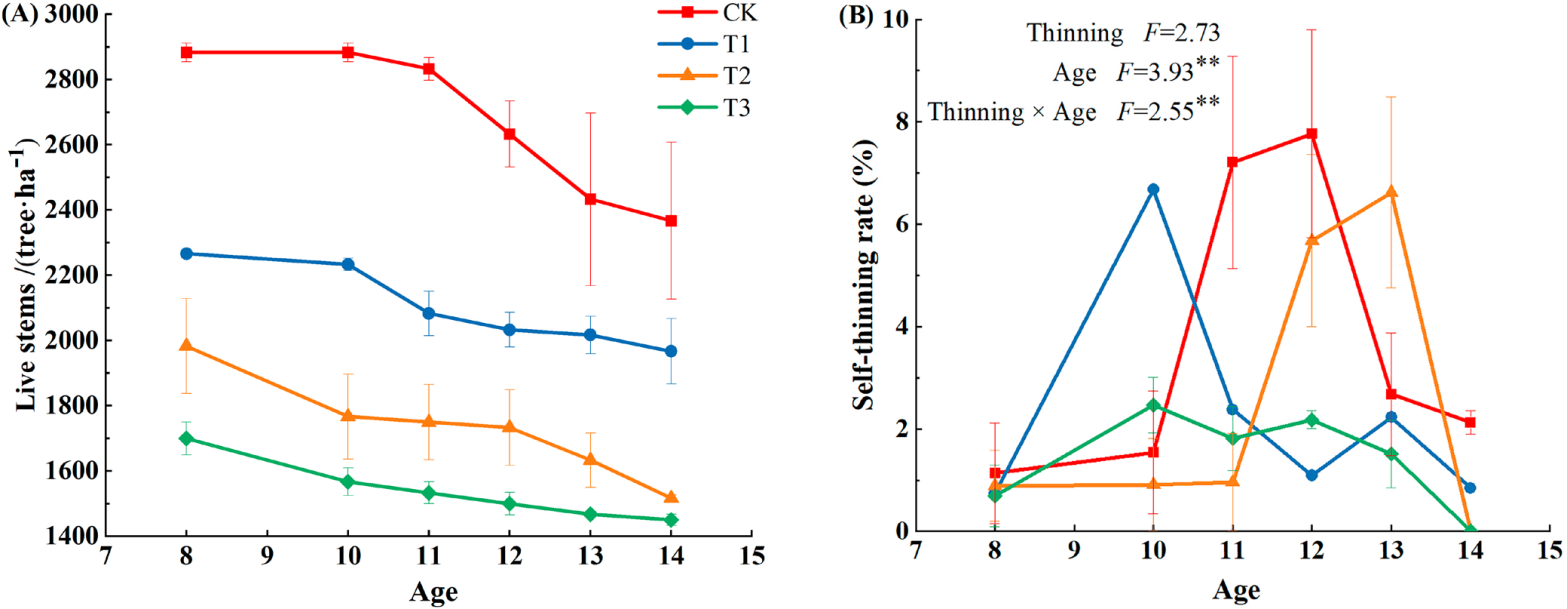
Number of living trees and self‐thinning rate in a *Cryptomeria japonica* plantation at four thinning intensities. CK: No thinning, T1: Light‐intensity thinning (20% of the trees removed), T2: Intermediate‐intensity thinning (30% of the trees removed), T3: Strong‐intensity thinning (40% of the trees removed). The lines represent the standard deviation of the means (*n* = 3).

The *Cryptomeria japonica* stand under study showed self‐thinning even after thinning was performed (Figure 2A). Furthermore, the number of living trees in CK plots decreased significantly with increasing stand age, reaching 2366 trees ha^−1^ at 8 years after establishment, compared to 2833 trees ha^−1^ (i.e., an 16.48% decrease) at the age of 14 years. During the 3–5 years after thinning, the number of standing trees decreased sharply, with a change rate of 16.56% in CK plots. Furthermore, the number of standing trees varied by approximately 6.7% within 2–3 years after thinning in T1 plots and remained stable thereafter. As for T2 and T3 plots, the variation in the number of standing trees was small regardless of stand age. Compared with the previous year, the number of standing trees of the same age gradually decreased with increasing thinning intensity. Although the number of surviving individuals in each treatment plot gradually tended to become constant after 14 years, the degree of size differentiation among individuals still showed a negative correlation with thinning intensity; in other words, the degree of size differentiation among individuals in the stand was also large at different thinning intensities, regardless of whether self‐thinning occurred.

Significant differences (*F* = 3.93, *p* < 0.01) among stands were observed for self‐thinning rate with increasing age (Figure 2B). In the first two years after thinning, the highest self‐thinning rate was 6.67% in T1 treatment plots, followed by 2.46% and 0.91% in T3 and T2 plots, respectively. However, the self‐thinning rate recorded in CK plots reached a maximum of 7.76% over the third to the sixth year after thinning, and 6.62% at five years after thinning in T2 treatment plots. In contrast, no changes in self‐thinning rate with increasing time were observed in T2 or T3 treatment plots at six years after thinning. Overall, stand age under high self‐thinning rate was between 10 and 13 years, indicating that individual competition within the stand was high and intense at this stage. Although no significant (*p* > 0.05) differences were observed among the four thinning intensities, the interaction between thinning and stand age had a significant (*p* < 0.05) effect on self‐thinning rate.

**FIGURE 3:**
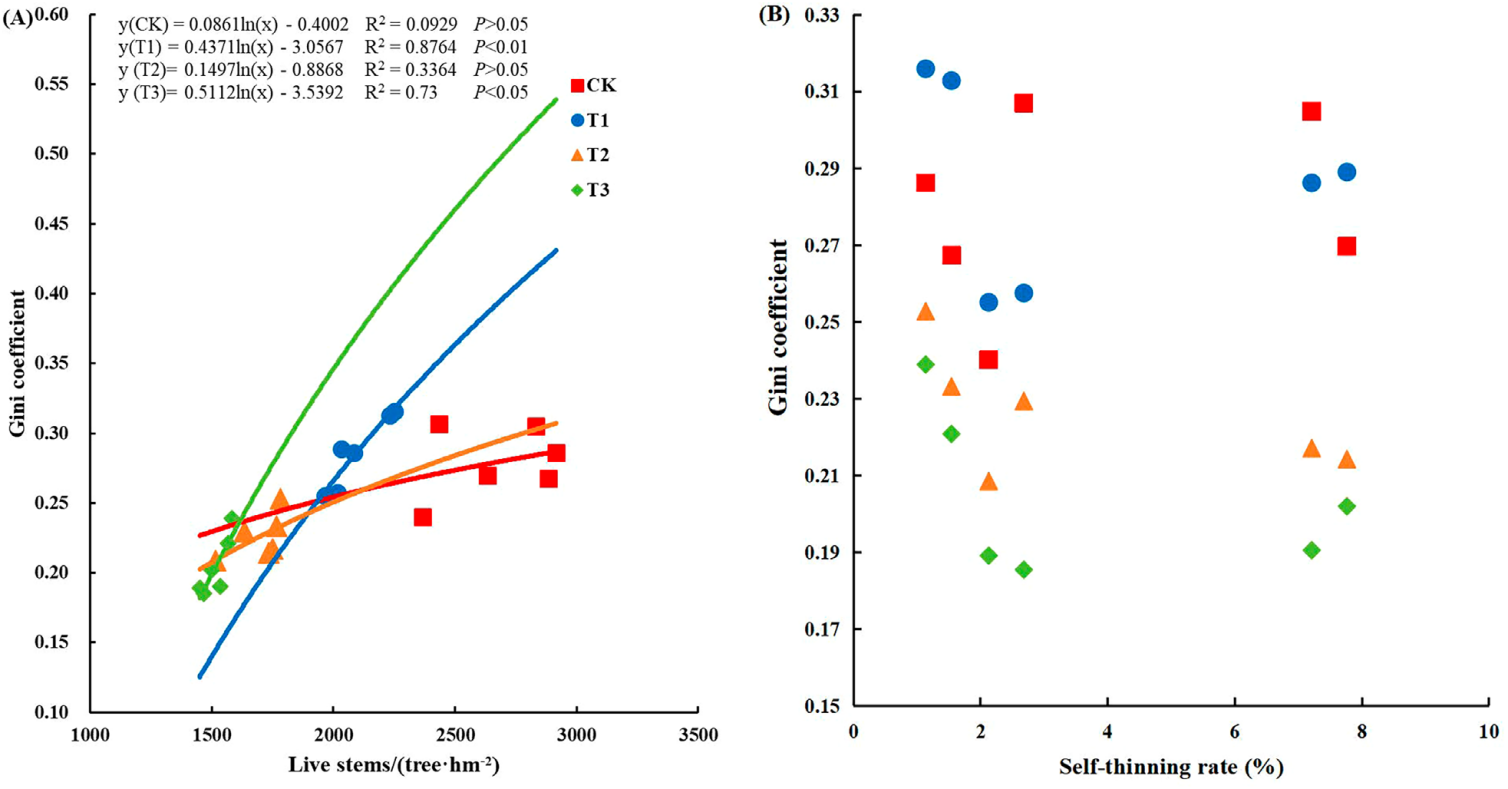
Relationship between Gini coefficient values and the number of living trees (A) and self‐thinning rate (B) under four thinning treatments. CK: No thinning, T1: Light‐intensity thinning (20% of the trees removed), T2: Intermediate‐intensity thinning (30% of the trees removed), T3: Strong‐intensity thinning (40% of the trees removed).

The relationship between Gini coefficient values and the number of living trees under the different thinning intensity treatments showed a logarithmic distribution, with the Gini coefficient increasing as the number of living trees increased (Figure 3A). In contrast, there was no significant correlation between self‐thinning rate and Gini coefficient values (Figure 3B).

**FIGURE 4:**
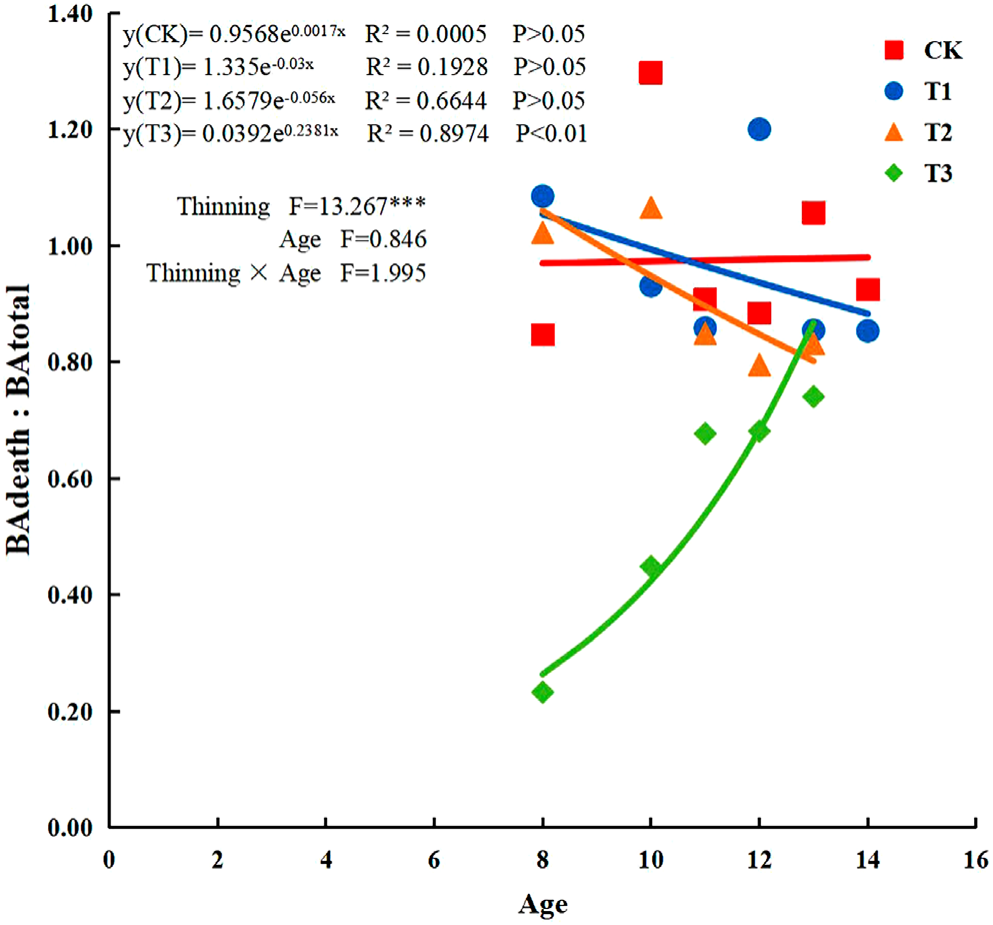
Ratio of the mean basal area of self‐thinning individuals per year to the mean basal area before stand death (BA_death_:BA_total_). CK: No thinning, T1: Light‐intensity thinning (20% of the trees removed), T2: Intermediate‐intensity thinning (30% of the trees removed), T3: Strong‐intensity thinning (40% of the trees removed).

During self‐thinning, the BA_death_:BA_total_ ratio increased significantly (*R* ^2^ = 0.8974, *p* < 0.01) with increasing stand age in T3 plots (Figure 4) but decreased in T1 and T2 plots. The BA_death_:BA_total_ ratio differed significantly (*F* = 13.267, *p* < 0.001) among the four thinning intensity treatments, and the interaction between stand age and thinning intensity had no significant effect. The distributions of BA_death_:BA_total_ and stand age were exponential functions under the four thinning treatments.

### 3.2 Size‐Class Distribution

**FIGURE 5:**
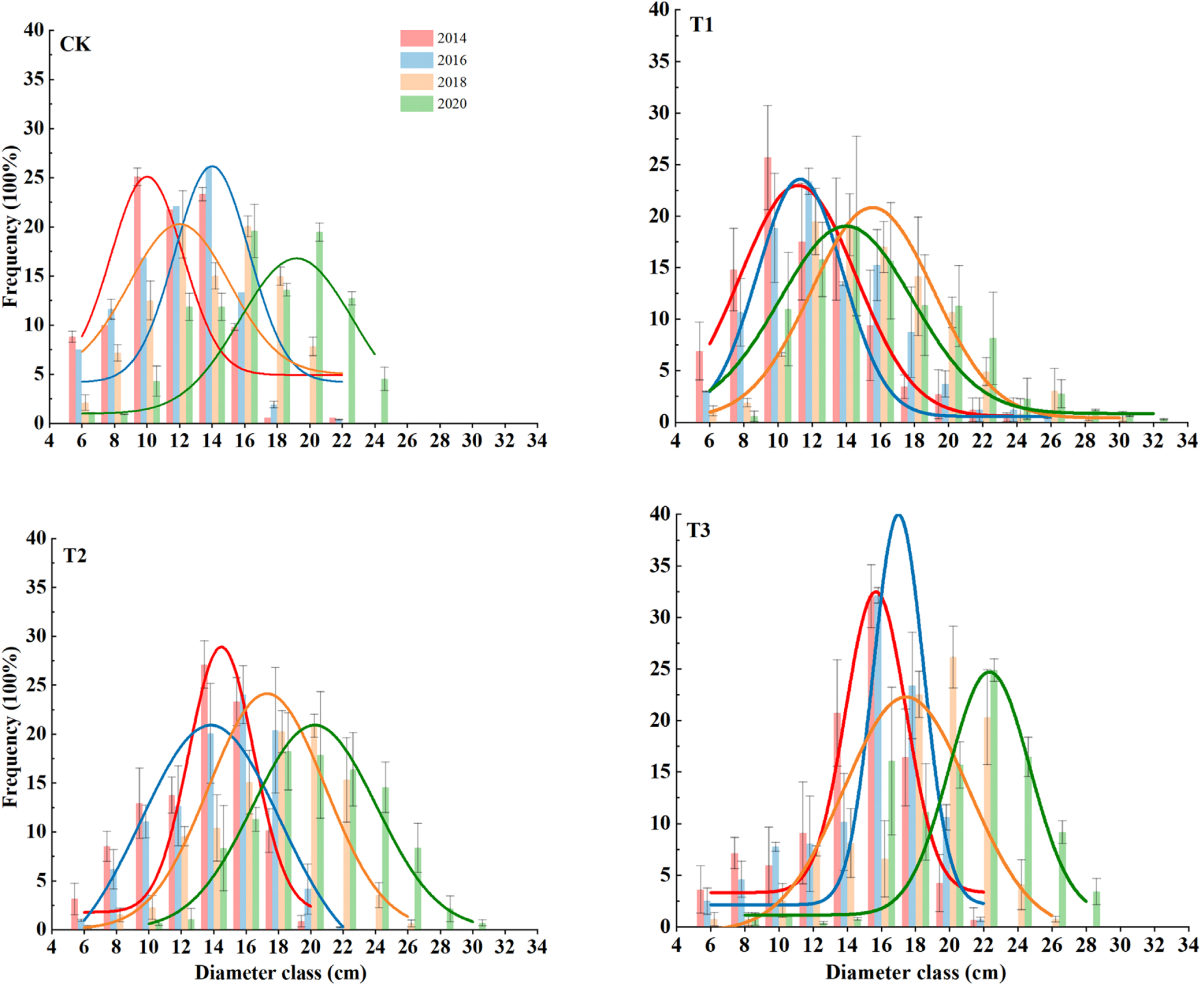
Diameter class distribution of frequency under four thinning treatments for the six‐year period from 2014 to 2020. CK: No thinning, T1: Light‐intensity thinning (20% of the trees removed), T2: Intermediate‐intensity thinning (30% of the trees removed), T3: Strong‐intensity thinning (40% of the trees removed). The bars represent the standard deviation of the means (*n* = 3).

Thinning enhanced diameter increase and altered diameter class‐frequency distribution. Thus, mean tree diameter in CK plots increased with increasing stand age, gradually shifting the normal distribution curve of the diameter class‐frequency distribution to the right and reducing kurtosis (Figure 5). In 2018, the normal distribution curve was between those for 2014 and 2016, with the diameter class‐frequency distribution in T1 ranging from 6 to 32 cm, in CK plots from 6 to 24 cm only, which was a significantly narrower range. As thinning time increased, the distribution curves gradually shifted to the right. After thinning, the proportion of the large‐diameter classes increased among three thinning intensities. Thus, in 2014, the largest mean diameters in CK, T1, T2, and T3 treatment plots were 10, 10, 14, and 16 cm, respectively, whereas in 2020, treatment mean diameter ranked, T3 > T2 > T1 > CK, with 22, 20, 14, and 16 cm, respectively.

**FIGURE 6:**
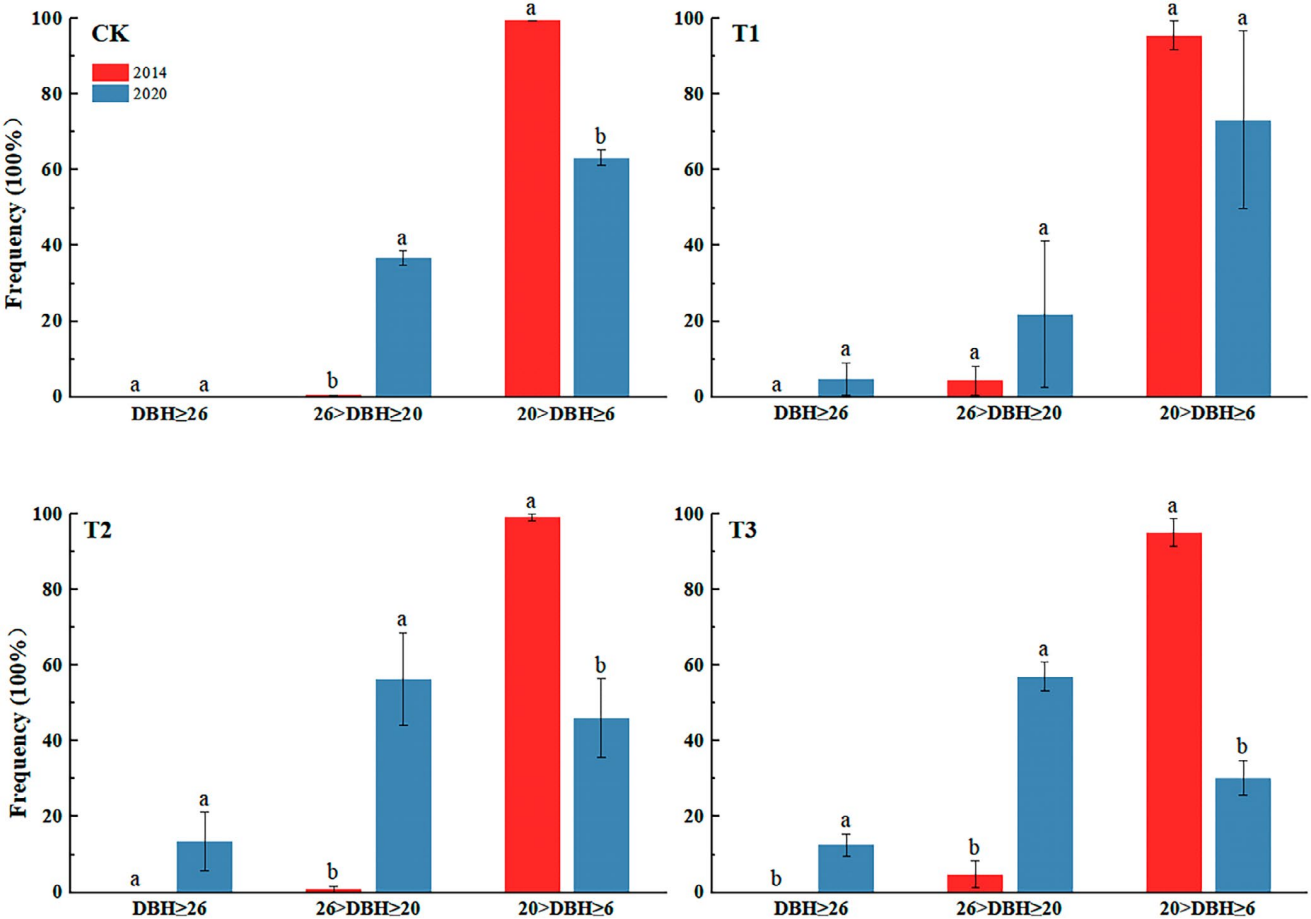
Large, intermediate, and small diameter frequencies under four thinning treatments in 2014 and 2020. CK: No thinning, T1: Light‐intensity thinning (20% of the trees removed), T2: Intermediate‐intensity thinning (30% of the trees removed), T3: Strong‐intensity thinning (40% of the trees removed). The bars represent standard deviation of the means (*n* = 3); different lowercase letters indicate significant differences of diameter frequency between 2014 and 2020 years in the same thinning intensities.

According to Figure 6, we clearly know that the large‐diameter (DBH ≥ 26 cm) were absent in each treatment in 2014, the small‐diameter (20 cm > DBH ≥ 6 cm) frequency was higher than others, and which were higher than 95%. 6 years after thinning, there is a clear change in the diameter of the large‐, intermediate‐, and small classes. In addition to CK treatment, the frequency of large diameter classes was 4.83%, 13.58%, and 12.62% in T1, T2, and T3 treatment, respectively. Both the CK and T1 treatments show a higher frequency in the small‐diameter class than in the intermediate‐diameter (26 cm > DBH ≥ 20 cm) class, while T2 and T3 show a larger frequency than in the intermediate‐diameter class.

### 3.3 Stand Productivity

**FIGURE 7:**
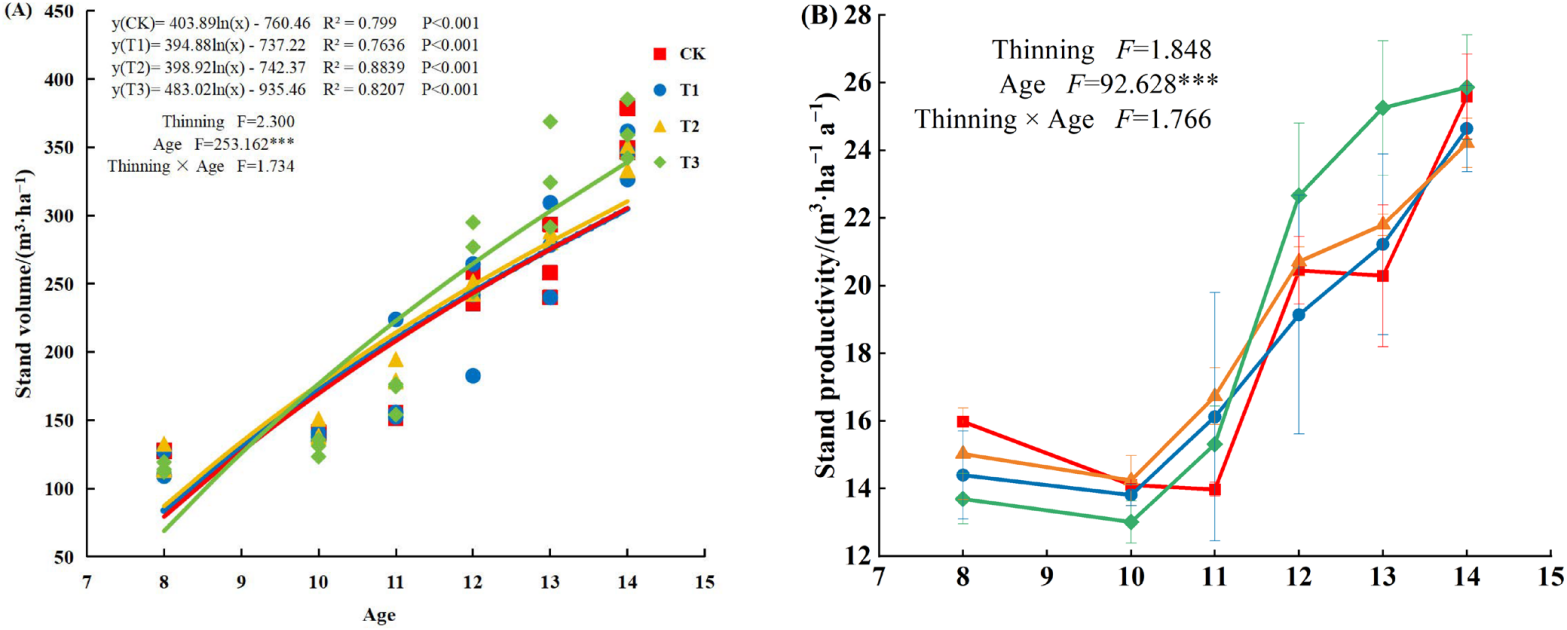
Stand volume and productivity of the experimental *Cryptomeria japonica* plantation under four thinning intensities. CK: No thinning, T1: Light‐intensity thinning (20% of the trees removed), T2: Intermediate‐intensity thinning (30% of the trees removed), T3: Strong‐intensity thinning (40% of the trees removed).

Although significant (*p* < 0.001) differences were observed in stand volume and productivity with stand age, neither thinning intensity nor the interaction between stand age and thinning intensity had any significant effect on stand volume or productivity (Figure 7). The distribution of stand volume changes with age was a logarithmic function for all thinning treatments, and stand volume increased with increasing stand age. The largest change in stand volume was recorded in T3 treatment plots, from 115.12 m^3^·ha^−1^ at 8 years to 362.07 m^3^·ha^−1^ at 14 years after plantation establishment, i.e., an increase of 246.95 m^3^·ha^−1^ over 6 years. T3 was followed by CK and T1 plots, in which stand volume increased by 230.50 and 229.67 m^3^·ha^−1^, respectively. In turn, T2 treatment plots showed the smallest increase in stand volume, at 218.83 m^3^·ha^−1^.

In general, stand productivity decreased first and then increased with stand age across thinning treatments. Maximum stand productivity was recorded when trees were 14 years old in each treatment. T3 and CK treatment plots showed higher productivity at 25.86 and 25.59 m^3^·ha^−1^, respectively, than those of T1 and T2 plots. Stand productivity at different thinning intensities showed different trends at different stand ages, among which stand productivity at 8–10 years in the CK treatment plots was the highest, followed by those of T2, T1, and T3. Moreover, although the stand productivity of the T3 treatment was the lowest at 8–11 years after establishment, it was the highest at 12–14 years, which was higher than that of any other thinning treatment.

**FIGURE 8:**
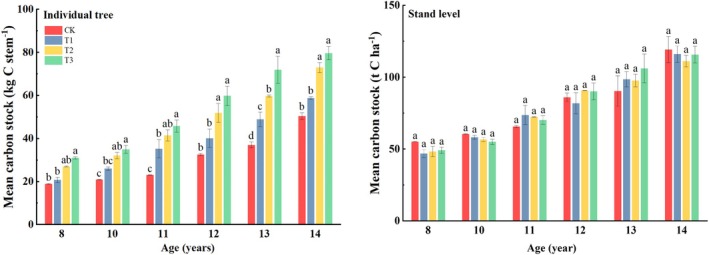
Carbon stock of four thinning treatments of the experimental *Cryptomeria japonica* plantation. CK: No thinning, T1: Light‐intensity thinning (20% of the trees removed), T2: Intermediate‐intensity thinning (30% of the trees removed), T3: Strong‐intensity thinning (40% of the trees removed). The bars represent the standard deviation of the means (*n* = 3); different lowercase letters indicate significant differences between different thinning intensities in the same index.

The carbon stock of individual trees increased with the increasing thinning intensity in each age stage, and the T3 treatment was significantly higher than the T1 and CK treatments, and the difference between T3 and T2 was significant only at 13 years old (Figure 8). From the perspective of the stand, although the carbon stock of the stand increased with time, there significant difference was absent among the thinning treatments in the same period. The carbon stock of the CK treatment was smaller than that of the thinning treatments at the age of 11–13 years and was larger than that of the thinning treatments at the ages of 8, 10, and 14 years.

## 4 Discussion

Individual differences in tree size reflect differences in acquisition and use of growth resources. Most studies have shown that the higher the initial planting density of a stand, the earlier the stand enters the competitive stage (Wallraf and Wagner 2019; Li, Liu, and Jin 2022), and the higher the degree of differentiation among individuals within the stand. Our study confirmed these findings, as shown by our observation that the degree of differentiation among individuals in the *C. japonica* sampled plots significantly decreased with increasing thinning intensity. Thus, the value for the Gini coefficient was highest in CK plots, followed by those for T1, T2, and T3 plots, indicating that higher stand densities maintained a higher level of stand differentiation.

A study conducted on *Eucalyptus* spp. showed that the Gini coefficient increased with stand age (Soares et al. 2016). However, after thinning, the Gini coefficient in the *C. japonica* plantation used in this study showed a negative correlation with stand age. This response may be related to stand age and stand density, as *Eucalyptus* trees used in that study were 2–8 year‐old and stand density ranged from 667 to 1667 tree·ha^−1^. Meanwhile, although *C. japonica* is a coniferous species that grows rapidly, its growth rate is relatively low compared to that of *Eucalyptus*. Additionally, because of the high initial planting density, self‐thinning in the *C. japonica* plantation used here was greater, reaching 16.48% in the CK treatment between tree ages of 8–14 (Figure 2A), resulting in a decrease in individual size differentiation with increasing stand age. The results of our study showed that the number of living trees decreased significantly with increasing stand age during self‐thinning in the *C. japonica* experimental plots, and the number of living individual trees gradually converged among the four thinning treatments, as demonstrated by the logarithmic association between individual size differentiation and the number of living trees. Regardless of whether self‐thinning occurred, the degree of individual‐size differentiation within the stand was large across thinning intensity treatments. In addition, we found that during self‐thinning, the BA_death_:BA_total_ ratio increased significantly with increasing stand age in T3 plots, whereas in T1 and T2 plots the ratio showed an overall decreasing trend while, concomitantly, both treatments showed values of the ratio BA_death_:BA_total_ > 1 in the first three years after thinning, when trees were 8–10 years old, suggesting that the average size of dead trees was larger than the size of the average tree in the entire stand as the stand increased with age during the self‐thinning process. High stand population density and basal area (BA) result in fewer resources per tree (Muller‐Landau et al. 2006), therefore the BA_death_:BA_total_ ratio increased significantly with increasing stand age in the T3 treatment, and the larger the tree, the more competitive it became (Luo and Chen 2011). BA_death_:BAtotal < 1 with increasing age suggests that the effect of dead trees on the entire stand decreased with increasing age. Consistently, previous studies showed that tree size significantly affects the death rate of smaller trees (Zhu et al. 2017), largely depending on nutrient and light availability, such that, when competition is intense, smaller trees struggle to maintain the carbon balance and have poor resistance, resulting in increased mortality. On the other hand, the surrounding tall trees overshadow smaller trees which, consequently, cannot obtain sufficient light. Studies have also shown that the tree mortality rate increases if the annual growth of the tree basal area fails to reach a threshold size before strong competition among trees within the stand begins (Qiu et al. 2015).

Stand diameter structure also changes with stand age (Kweon and Comeau 2019). By removing suppressed, radial growth‐limited trees, thinning can improve light incidence within the canopy and soil nutrient availability, thereby promoting the radial growth of the remaining trees. In addition, the removal of small trees further promotes the growth of medium and large trees, leading to significant changes in stand diameter structure (Willis et al. 2018). In this study, thinning accelerated the differentiation of the diameter class‐frequency distribution. Thinning reduced the left skewedness of the diameter class‐frequency distribution curve of the stand structure, with that of treatment T1 being the most significant case, in which the diameter class‐frequency distribution curve was right‐skewed, and the peak was gradually reduced. The diameter class‐frequency distribution curve was significantly right‐skewed under T2 and T3 treatments in 2020. These results are consistent with those of previous studies on *Cunninghamia lanceolata* (Li et al. 2023), which indicated that as thinning intensity increased, the diameter class‐frequency distribution of the sample stands moved rightward. According to the frequency of the large‐medium‐small diameter classes, thinning significantly increases the large diameter (DBH ≥ 26 cm), especially in the T3, T2 treatments, which more benefit the larger diameter cultivation. Thinning alters the space of individual trees and the microenvironment, providing better living conditions; thus, higher thinning intensities increase the large diameter compared to the CK and T1 treatments.

The significant relationship between stand volume and stand age in the *C. japonica* plantation in this study followed a logarithmic pattern. Early in the growth cycle of the stand, the proportion of intermediate‐diameter timber was higher than that recorded when a quantitatively mature stand was reached, although the intensity of competition between individuals within the stand increased with increasing stand age. However, the removal of small‐ and intermediate‐diameter trees resulted in a reduction in the total number of trees in the stand concomitant with an increase in mean DBH. *C. japonica* 8–14‐year‐old trees experienced a fast‐growing period, and the results showed the greatest increase in stand volume in T3 treatment plots, followed by that in CK plots, while the smallest volume change was characteristic of T2 treatment plots at 6 years after thinning. In the 14‐year‐old *C. japonica* stand, trees under treatment T3 showed the greatest change in DBH diameter and the greatest number of trees in the large diameter classes (Figure 5), although the total number of trees per plot in the stand was the lowest. Conversely, the number of trees played a dominant role in the CK treatment plots of the stand.

Some previous studies have shown that, although thinning can promote single‐tree volume while increasing the proportion of intermediate‐ and large‐sized timber, it cannot increase stand volume (Mäkinen and Isomäki 2004; Bose et al. 2018), as confirmed by treatments T1 and T2 in this study, mainly owing to the fact that the shift from high to low stand density reduced the intensity of intraspecific competition, and the retained trees had access to sufficient growing space and nutrient resources. In addition, the average annual volume (stand productivity) of *C. japonica* stands of different ages showed fluctuating changes with increasing stand age, and the productivity was highest in 8–10‐year‐old stands under the CK treatment, presumably because the effect of thinning (i.e., treatments T1, T2, and T3) was not yet apparent at that time. In contrast, the productivity of the T3 treatment was higher than that of any other treatment in the 11–14‐year‐old stands and showed a stable trend in the 13–14‐year‐old ones, mainly because trees were in the rapid growth stage during the thinning period and rapid accumulation of biomass at the time led to a rapid increase in the level of stand productivity. One research study about *Larix principis‐rupprechtii* showed that the productivity per ha per year was higher in the heavy thinning (Li et al. 2020).

Although the carbon stock of individual trees and stand increased with time, the individual trees appear obviously trendy with thinning intensities (Figure 8). In the whole, at the age of 11–13 years, stand stock of CK treatment was lower than thinning treatment, while in the years of 8, 10, and 14, it was higher than thinning treatment. Although we have previously mentioned that thinning increased the proportion of large‐intermediate diameter classes and the productivity of *Cryptomeria japonica* , the number of trees decreased after thinning and the growth rate does not make up for the missing amount. Although there was a clear trend of change between treatments during our study period, this needs to be continuously monitored.

## 5 Conclusions

Through a study of individual differentiation characteristics, diameter class‐frequency distribution, stocking volume evolution, and carbon stocks in *C. japonica* stands under four thinning intensities were characterized. We found that the Gini coefficient decreased with increasing thinning intensity and stand age. The number of living individual trees in each treatment gradually converged, and the degree of size differentiation between individuals showed a negative correlation with thinning intensity. Additionally, the mean diameter at breast height in each treatment increased with increasing stand age, and the normal distribution curve of diameter class frequency gradually shifted to the right, with slight changes in the CK treatment and the largest in the T3 treatment plots. Higher thinning intensities had a higher frequency of large‐diameter (DBH ≥ 26 cm) timber. Stand volume and productivity in the *C. japonica* stands used in this study varied with stand age, with the greatest change in stand volume occurring under treatment T3. In summary, for 8‐year‐old *C. japonica* plantation, 30%–40% (T2 and T3) thinning intensity can be selected; the stand volume and carbon stock of individual trees increased with increasing thinning intensity and time, which is conducive to the growth of large‐diameter timber. In addition to this, we suggest that secondary thinning can occur after 6 years, as the self‐thinning rate of T1 treatment is still occurring. The paper studied tree differentiation and productivity and carbon stock of a *C. japonica* plantation under thinning to devise proper management. However, the applied buffer zone of 5 m between plots should be considered too small to reduce potential edge effects, which is a potential limitation of this study. In addition, more attention should be paid to long‐term monitoring and the cumulative effects of thinning treatments on tree growth and differentiation, as well as wood quality.

## Author Contributions

**Kaili Liu:** conceptualization (equal), data curation (equal), formal analysis (equal), investigation (equal), methodology (equal), software (equal), writing – original draft (equal), writing – review and editing (equal). **Boyao Chen:** data curation (equal), investigation (equal), visualization (equal). **Bin Zhang:** formal analysis (equal), investigation (equal), software (equal). **Pu Zhou:** formal analysis (equal), investigation (equal), software (equal). **Ruihui Wang:** conceptualization (equal), funding acquisition (equal), methodology (equal), supervision (equal). **Chunsheng Wang:** formal analysis (equal), methodology (equal), software (equal), supervision (equal), writing – review and editing (equal).

## Conflicts of Interest

The authors declare no conflicts of interest.

## Supporting information


Data S1.

## Data Availability

Data are provided as a “Supporting Information” file.
